# Nurses, Their Uniform and the Public

**Published:** 1895-09-21

**Authors:** 


### The Hospital

An Institutional Journal of

The Medical Sciences and Hospital Administration, with

AN EXTRA NURSING SUPPLEMENT.

Vol. XVIII.—No. 469.] [Sept. 21, 1895.

### Nurses, Their Uniform and the Public.

During the last few weeks complaints have been made in the public press that the manager of a West End hotel refused to admit a nurse in uniform as a guest. This refusal has called forth a series of remonstrances from certain members of the nursing profession, who feel themselves aggrieved by the regulation in question. We have made a careful investigation into the whole of the circumstances surrounding the admission of nurses in uniform to popular hotels in London and elsewhere. We find that this question presents difficulties of various kinds which are not appreciated either by the nurses or the public. Naturally the hotel proprietor, for the purposes of his business, desires to make such regulations only as shall enable him to conduct his house to the satisfaction of his guests, as his profits are dependent upon the success or otherwise with which his efforts are crowned. It follows that he would not knowingly take any step likely to prove unpopular, or calculated to give offence to his regular customers. Hence, it may be taken for granted that the evidence must have pointed very strongly in a certain direction, or no manager would have declined to admit a nurse in uniform as a guest.

We believe that the root of the whole matter rests in the difficulty presented by the growing abuse of the use of uniform by women who are not nurses at all. Experience has created a strong impression in the minds of hotel keepers and others that more and more women who are not nurses affect some kind of uniform, because it is supposed to be more attractive to men than ordinary dress. This being so, we have on the one hand the legitimate nurse on duty with a patient who desires to stay at a particular hotel, and on the other hand the impostor, also in uniform, who desires to reside in an hotel for her own private reasons. If we had only these two classes to deal with, the difficulty of abolishing the evil would not be insurmountable. Unfortunately there are a certain number of nurses entitled to wear uniform to be met with occasionally in hotels who are inclined to hang about the corridors, and in other ways to make themselves objectionable to the management and to the guests. This intermediate class of women forms the connecting link between the *boná fide* nurse inspired with a high sense of the honour of her profession and the impostor who trades upon a uniform to which she has no claim. We are bound, therefore, to say that if nurses desire to be welcome guests at hotels, they, or the best of them, must combine to put down the flighty members of their own body, who form the connecting link between themselves and the impostors as already explained.

So far as the hotel keepers are concerned we have ascertained that the general practice is not to refuse to admit a nurse in uniform as a guest. There are, however, hotels and hotels. Some hotels like those in London are mainly houses of call frequented by travellers and others, where the presence of a nurse in uniform is a matter of comparative indifference to the busy throng of guests who come in and out every day in the week. Here, so far as we have been able to ascertain—that is, in the largest and best hotels in London—no objection has been raised to *boná fide* nurses in uniform accompanying guests. The case of a great hotel like the Metropole at Brighton, which owes its success mainly to the fact that it is conducted and managed on principles which promote amusement and the enjoyment of pleasure only, is wholly different. At such an hotel the presence of anything unpleasant, even by suggestion, is to be avoided, because everybody who goes there goes in search of pleasure, relaxation, and amusement. Hence nurses in uniform in attendance upon sick people are there quite out of place. Besides, no sensible patient recovering from illness would venture to select this class of hotel as a place calculated to suit his invalid condition. All the surroundings of such an establishment are ill calculated to promote recovery or to secure the comfort of a convalescent. But the woman who masquerades in nurse's uniform for her own private purposes would prefer, if she could, to obtain admission to an hotel of this description. All these facts are fully recognised and understood by the managers of hotels generally, and we commend them to the attention of nurses, the medical profession, and the public, because the better they are understood the less likelihood will there be that any difference of opinion can arise in regard to the wearing of uniform in places of public resort.

The hotel keepers do not, as we have stated, refuse as a general practice to admit nurses wearing uniform as guests, but when, as occasionally happens, the wearers of nurses' uniform at at any hotel amount to so large a number as to attract the attention of the other guests, or to call for their remonstrance, then the hotel keepers in self-defence are compelled to request all the wearers to assume ordinary dress so long as they remain there. Such a request must prove quite unobjectionable in hotels like the Metropole, Brighton, when all the circumstances are borne in mind. A nurse is only called upon to wear uniform when she is on duty. In purely pleasure hotels no nurse will be found on duty, because they are not suited either from the patient's or from the visitors' point of view to the reception of sick people who require the attendance of a trained nurse. In all other cases nurses in uniform who conduct themselves with discretion may enter an hotel as a guest without objection, providing steps are taken on behalf of the nursing profession to institute proceedings against every woman proved to be wearing nurse's uniform who is not entitled to do so. We would therefore suggest that this question is now in a condition to be taken up by the Royal British Nurses' Association, which might thus give sureties to the profession and the public that it intends to represent the interests of nurses whenever such representation will conduce to the welfare of the public and the nursing body as a whole. If an association of nurses is ever to be of practical utility, it must take charge of questions of this kind, and that is why we hope that the Royal British Nurses' Association will approach the hotelkeepers and secure that in future no woman can masquerade in nurse's uniform without running the risk of prosecution and conviction.

